# Vitamin D Serum Status and Associated Factors Among Women with Cervical Lesions

**DOI:** 10.3390/epidemiologia7020040

**Published:** 2026-03-04

**Authors:** Zinhle Simelane, Likhona S. Masika, Charles B. Businge, Zizipho Z. A. Mbulawa

**Affiliations:** 1 Department of Laboratory Medicine and Pathology, Walter Sisulu University, Mthatha 5100, South Africa; 218049412@mywsu.ac.za (Z.S.); lmasika@wsu.ac.za (L.S.M.); 2 Department of Obstetrics and Gynaecology, Walter Sisulu University, Mthatha 5100, South Africa; cbusinge@wsu.ac.za; 3 National Health Laboratory Service, Nelson Mandela Academic Hospital, Mthatha 5100, South Africa

**Keywords:** serum vitamin D, women, abnormal cervix, body mass index, human immunodeficiency virus

## Abstract

Background/Objectives: Vitamin D plays a role in cellular regulation and immune processes relevant to cervical carcinogenesis, yet data on vitamin D status and its determinants in high-burden settings such as South Africa remain scarce. This paper therefore describes the prevalence of vitamin D deficiency, insufficiency, and sufficiency, and explores associated factors among women with cervical lesions. Methods: A descriptive cross-sectional study was conducted among 103 women aged 18–81 years. Women were referred to Nelson Mandela Academic Hospital due to cervical cancer, high-grade squamous intraepithelial lesions (HSILs), or atypical squamous cells—cannot exclude HSIL, or low-grade squamous intraepithelial lesions, or atypical squamous cells of undetermined significance. The total serum 25(OH)D (D_2_ + D_3_) was quantified using the MAGLUMI 25-OH Vitamin D chemiluminescent immunoassay kit on the MAGLUMI X3 Fully Automatic Chemiluminescence Immunoassay Analyzer (Snibe Diagnostic, Shenzhen New Industries Biomedical Engineering Co., Ltd., Shenzhen, China). Serum vitamin D was categorized according to the Endocrine Society Task Force guidelines. Results: Vitamin D insufficiency was observed in 46.60% of participants and deficiency in 26.21% while only 27.18% had sufficient levels. Overall, vitamin D deficiency or insufficiency was more common than sufficiency (72.82%; 27.18%, *p* < 0.0001). Among HIV-positive women, 78.26% had vitamin D deficiency or insufficiency compared with 63.33% of HIV-negative women; however, this difference was not statistically significant. Vitamin D deficiency was most prevalent in women with healthy body mass index (BMI, 46.40%) values and decreased significantly with increasing BMI values (*p* = 0.008). Conclusions: Vitamin D deficiency and insufficiency were common among women with cervical lesions in this rural South African population. Associations with BMI suggest context-specific influences on vitamin D status. Owing to the study’s cross-sectional design and lack of normal cervical cytology participants, the findings are descriptive and exploratory, underscoring the need for longitudinal and comparative research to better define the role of vitamin D in cervical disease.

## 1. Introduction

While vitamin D is classically associated with calcium and phosphate homeostasis, growing evidence indicates that it also plays an important role in non-calcemic biological processes relevant to cancer development. In its active form, calcitriol (1,25-dihydroxyvitamin D), vitamin D, influences gene expression via the vitamin D receptor (VDR), thereby affecting cellular growth, differentiation, immune responses, and programmed cell death [1,2]. Beyond these genomic effects, vitamin D also exhibits anti-angiogenic and anti-metastatic properties in several tumour models, though the magnitude of these effects varies by cancer type and disease stage [2]. In cervical-cancer experimental systems, exposure to 25(OH)D_3_ has been shown to reduce HeLa cell viability, promote apoptosis, and up-regulate enzymes of the vitamin-D-metabolising pathway, suggesting that cervical epithelial cells can locally synthesize and respond to vitamin D [3,4].

It is generally estimated that in humans, around 80% of vitamin D comes from ultraviolet-B (UVB)-driven production in the skin while food sources contribute only a small portion [5]. Endogenous vitamin D production can be substantially reduced by several key factors that contribute to deficiency. These include geographical influences, such as residence at latitudes beyond 40° during winter months; socio-cultural determinants, including clothing habits; the use of high-sun-protection-factor sunscreens; inadequate dietary practices (including poorly planned vegan diets); and lifestyle patterns that limit outdoor exposure such as predominantly indoor occupations, exercise routines confined to indoor facilities, extended working hours, urbanization, migration from African or Asian regions to Western countries, and religious practices involving extensive body covering; as well as physiological determinants, including increased skin pigmentation, older age, obesity, chronic health conditions, and certain genetic polymorphisms [6,7,8]. Additionally, numerous medications influence the vitamin D metabolic pathways, thereby altering an individual’s overall vitamin D requirements [8]. The drug categories known to affect serum 25(OH)D concentrations are reviewed in detail in other sources [8,9].

Cervical cancer ranks among the leading cancers affecting women worldwide and continues to pose a significant public health challenge, with an especially high burden observed in Sub-Saharan Africa [7,10]. Each year in South Africa, cervical cancer accounts for roughly 11,000 newly identified cases and close to 5900 deaths, placing it among the most prominent contributors to cancer-related mortality in the female population [11,12]. The etiologic driver is persistent infection with oncogenic human papillomavirus (HPV) types [13,14]. Nevertheless, infection with HPV by itself does not inevitably lead to the development of high-grade lesions or invasive cancer. Instead, progression is influenced by a range of host-related factors, such as immune function, nutritional status, concurrent infections (including human immunodeficiency virus), and cellular regulatory pathways governing proliferation and apoptosis, which together shape the transition from HPV infection to cytological abnormalities and more advanced disease [15].

Considering the potential impact of vitamin D on cervical lesions and that vitamin D deficiency is prevalent among black women compared to white women worldwide, the distribution of vitamin D needs to be examined in South African rural settings. To date, only a handful of epidemiological studies have explored vitamin D levels and associated factors in women with cervical lesions [14,16]. Therefore, this study investigated the prevalence of vitamin D sufficiency, insufficiency, and deficiency, along with associated factors, among South African women with cervical lesions.

## 2. Materials and Methods

### 2.1. Study Design, Setting, Population

The study employed a descriptive cross-sectional design. Women referred to Nelson Mandela Academic Hospital’s obstetrics and gynecology department due to cervical cancer, high-grade squamous intraepithelial lesions (HSILs), atypical squamous cells—cannot exclude HSILs (ASC-H), low-grade squamous intraepithelial lesion (LSILs), or atypical squamous cells of undetermined significance (ASC-US) who met the inclusion criteria were recruited. As a tertiary teaching and referral institution, Nelson Mandela Academic Hospital delivers specialized healthcare services to populations residing in the OR Tambo district and parts of the Chris Hani, Alfred Nzo, Joe Gqabi, and Amathole district municipalities in the Eastern Cape Province, South Africa.

Eligibility was restricted to women aged 18 years or older who were referred with a diagnosis of cervical cancer or abnormal cervical cytology, including HSIL, ASC-H, LSIL, or ASC-US. Participants were required to be non-pregnant, and not menstruating or experiencing active vaginal bleeding at the time of enrollment, and to confirm prior sexual activity. All cervical cancer diagnoses were confirmed by histopathological evaluation. For participants with cytology-based diagnoses of HSIL, ASC-H, LSIL, or ASC-US, corresponding histopathological records were unavailable for most cases; consequently, analyses were conducted using cytological findings only. No additional or independent morphological evaluation was conducted beyond the original diagnostic assessment.

Data was collected through confidential, interviewer-administered sessions conducted in participants’ preferred languages, with careful consideration of cultural sensitivity and confidentiality. Structured questionnaires were used to obtain information on socio-demographic characteristics, alcohol consumption, tobacco use, sexual practices, gynecological history, vitamin D supplementation, and exposure to recognized dietary and environmental sources of vitamin D. Standardized anthropometric measurements were obtained for all participants, including height (m) and body mass (kg), from which body mass index (BMI) was subsequently calculated. Each participant’s Human Immune Virus (HIV) serostatus was documented at the time of clinical assessment. Individuals with undocumented or previously negative HIV results were offered voluntary point-of-care testing administered by qualified nursing personnel. Newly identified HIV infections were managed in accordance with prevailing South African national HIV treatment protocols. For laboratory analyses, approximately 5 mL of peripheral venous blood was collected from each participant by trained nursing staff and processed using serum separator tubes (Becton, Dickinson and Company, Franklin Lakes, New Jersey, NJ 07417, USA).

### 2.2. Sampling Frame and Sample Size

This was a descriptive cross-sectional study in which women with abnormal cervical cytology and a histologically confirmed diagnosis of cervical cancer referred to Nelson Mandela Academic Hospital were recruited between June and August 2025. The sample size was calculated using the single population proportion formula, $n=\frac{Z^{2}\times P(1-P)}{d^{2}}$, with a 95% confidence level and 10% margin of error. An estimated prevalence (P) of approximately 28.5% for vitamin D deficiency in the Eastern Cape Province was used, based on a previous cross-sectional study [17]. Using these assumptions, the minimum required sample size was estimated at 90. Ultimately, recruitment surpassed this target, resulting in a final sample of 103 participants.

### 2.3. Study Variables

The primary outcome variable was serum vitamin D status, analysed both as categorical variable (deficient, insufficient, sufficient) and dichotomised (<20 ng/mL, 20–30 ng/mL, and >30 ng/mL). Independent variables included age, marital status, education level, employment status, monthly income, BMI, HIV status, smoking and alcohol use, reproductive history, skin complexion, self-reported sun exposure, vitamin-D-containing food, and abnormal cervical cytology/histology category.

### 2.4. Laboratory Investigations

The blood specimen in the serum separator tube was centrifuged at 3500 rpm for 10 min within three hours of specimen collection. The specimens were stored at 4 °C and analyzed within five days of collection. Short-term storage at this temperature is appropriate for preserving the stability of serum 25-hydroxyvitamin D prior to analysis. The total serum 25(OH)D (D_2_ + D_3_) was quantified using the MAGLUMI 25-OH Vitamin D chemiluminescent immunoassay kit (Snibe Diagnostic, Shenzhen New Industries Biomedical Engineering Co., Ltd., Shenzhen, China) on the MAGLUMI X3 Fully Automatic Chemiluminescence Immunoassay Analyzer (Snibe Diagnostic, Shenzhen New Industries Biomedical Engineering Co., Ltd., Shenzhen, China). Endocrine Society Task Force’s 25(OH)D cut-off point recommendations were employed, which classified vitamin D levels as deficient (<20 ng/mL), insufficient (20–30 ng/mL), and sufficient (>30 ng/mL). Assay performance was monitored using daily internal quality control measures and verified through external quality assurance participation, with results consistently meeting established standards for precision and accuracy.

### 2.5. Statistical Analysis

All variables obtained from questionnaires and laboratory analyses were coded and entered into Microsoft Excel version 2016 (Redmond, WA 98052, USA) and subsequently imported into SPSS Statistics version 30 (IBM Corp., Armonk, NY, USA) for analysis. Continuous variables were assessed for normality using the Shapiro–Wilk test and visual inspection of histograms. Non-normally distributed variables were summarized using medians and interquartile ranges (IQRs), and group comparisons were performed using the Wilcoxon rank-sum test. Categorical variables were summarized using frequencies and percentages. Differences in proportions between groups were assessed using the two-sample test of proportions. Associations between categorical variables were evaluated using the chi-square test or Fisher’s exact test, with Fisher’s exact test applied when ≥20% of expected cell counts were <5 or when any expected cell count was zero.

Given the high prevalence of vitamin D deficiency and insufficiency in the study population, prevalence ratios (PRs) were estimated using contingency table analyses rather than logistic regression, as odds ratios would overestimate associations. Due to the limited sample size, multivariable regression modeling (e.g., Poisson regression with robust variance) was not performed. Consequently, all PRs presented are crude (unadjusted) and should be interpreted cautiously. Statistical significance was defined at a two-sided *p*-value ≤ 0.05, and all estimates are presented with 95% confidence intervals (CIs).

## 3. Results

### 3.1. Study Population Description

A total of 103 participants between the ages of 18 and 81 were enrolled in the study. The study population was predominantly comprised of women aged between 41 and 60 years, making up 53.40% of the study population. The majority of the participants were unmarried or single (53.40%, 55/103), had a high-school education level (44.66, 46/103), were not currently employed (84.47, 87/103), had a monthly income of ZAR 1000–ZAR 3999 (69.90%, 72/103), had been pregnant three or more times (70.87%, 73/103), were HIV-positive (66.99%, 69/103), and had been referred to hospital due to HSIL/ASC-H (59.22%, 61/103). All the HIV-positive women were on antiretroviral therapy (ART). Regarding lifestyle behaviors, a substantial proportion (85.44%, 88/103) reported never having consumed alcohol, whereas 6.80% (7/103) indicated past alcohol use. Similarly, only about 7.77% (8/103) of the participants were current smokers, while 91.26% (91/103) reported that they had never smoked in their lifetime. The study further assessed the sexual and reproductive health profiles of the participants, in which the median age at sexual debut was 18 years (IQR: 16–20) and they had a median of three lifetime sexual partners with an interquartile range between two and five (Table 1). None of the study participants reported a current use of vitamin D supplementation.

### 3.2. Serum Vitamin D Levels Among Women with Cervical Lesions

Across the study population, vitamin D deficiency and insufficiency were highly prevalent, collectively affecting 72.81% (75/103) of women. Specifically, 26.21% (27/103) of the participants exhibited vitamin D deficiency (<20 ng/mL), while 46.60% (48/103) demonstrated insufficiency (20–30 ng/mL). Only 27.18% (28/103) had sufficient vitamin D levels (>30 ng/mL), underscoring a significant burden of vitamin D insufficiency in this population. Among HIV-negative participants, 26.70% were vitamin-D-deficient, and 36.70% had insufficient levels, resulting in 63.40% having suboptimal levels (≤30 ng/mL). In contrast, HIV-positive participants demonstrated a slightly higher prevalence of insufficiency 52.17% (36/69) and a comparable prevalence of deficiency (26.09%), yielding a total of 78.21% with low vitamin D status. HIV infection did not have a significant impact on the observed prevalence between the vitamin D categories. In the overall study population, a greater proportion of women had vitamin D deficiency or insufficiency compared with sufficient levels (72.82%, 75/103; 27.18%, 28/103, *p* < 0.0001). This finding was also observed among HIV-positive women (78.26%, 54/69; 21.74%, 15/69). However, among HIV-negative women, the difference between deficiency/insufficiency and sufficiency was not statistically significant (63.33%, 19/30; 37.67%, 11/30; *p* = 0.070, Table 2).

### 3.3. Factors Associated with Vitamin D Deficiency Among Women with Cervical Lesions

Women with a monthly income of ≥ZAR 1000–3999 had a lower prevalence of vitamin D deficiency compared with those earning < ZAR 1000; however, this association was borderline and did not reach conventional statistical significance (PR = 0.43; 95% CI: 0.23–0.97; *p* = 0.056). A significantly higher proportion of women with a normal BMI had vitamin D deficiency than obese women (PR: 0.35, 95% CI: 0.16–0.75, *p* = 0.008, Table 3). The prevalence of vitamin D deficiency decreased with an increasing age and increased among individuals aged 61–81 years. When comparing the age group 51–60 years with 61–81 years’ women, there was a 92% chance of vitamin D deficiency among individuals aged 61–81 years (PR: 1.92, 95% CI: 0.66–6.13, *p* = 0.309); however, this was not statistically significant. Women who self-reported having a dark skin complexion had a 73% higher chance of vitamin D deficiency than light-skinned women (PR: 1.73, 95% CI: 0.81–3.91, *p* = 0.234). Women with a history of vaginal itchiness and/or discharge had a 42% chance of vitamin D deficiency, and those with a history of genital ulcer/warts had a 70% chance of vitamin D deficiency compared to those with no history. A different abnormal cervical cytology/histology status was not associated with vitamin D deficiency (Table 3).

### 3.4. The Distribution of Vitamin D Levels by BMI Status Among Women with Cervical Lesions

**Figure 1:**
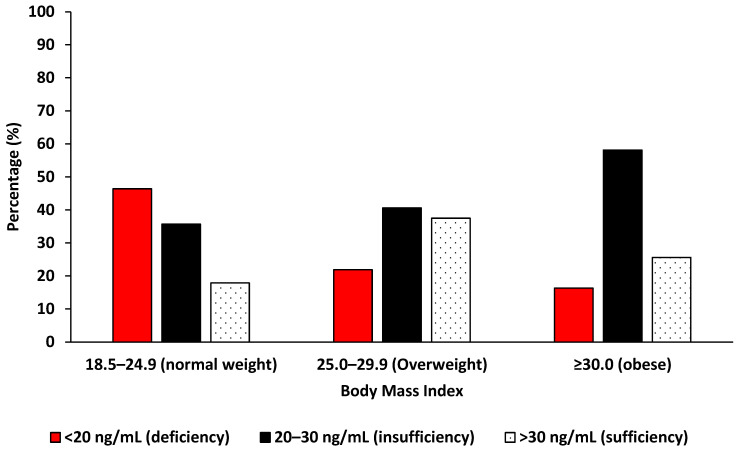
The distribution of vitamin D levels by body mass index among Eastern Cape women with cervical lesions.

Vitamin D deficiency was observed to be common among women with a normal BMI than obese women (PR 0.35, 95%CI: 0.16–0.75, *p* = 0.008, Table 3), affecting 46.43% (13/28), and its prevalence significantly decreased with an increasing BMI (*p* for trend = 0.008), with obese women showing the lowest deficiency rate at 16.27% (7/43). In contrast, an increasing pattern of vitamin D insufficiency was observed with higher BMI, rising from 35.71% (10/28) among normal weight women to 58.14% (25/43) among obese women, though the increase was not statistically significant and only showed a borderline trend (*p* for trend = 0.053). Vitamin D sufficiency was lowest in the normal BMI group, increased among overweight women, and slightly declined in obese women, with no significant trend across BMI categories (*p* for trend = 0.603, Figure 1).

**Figure 2:**
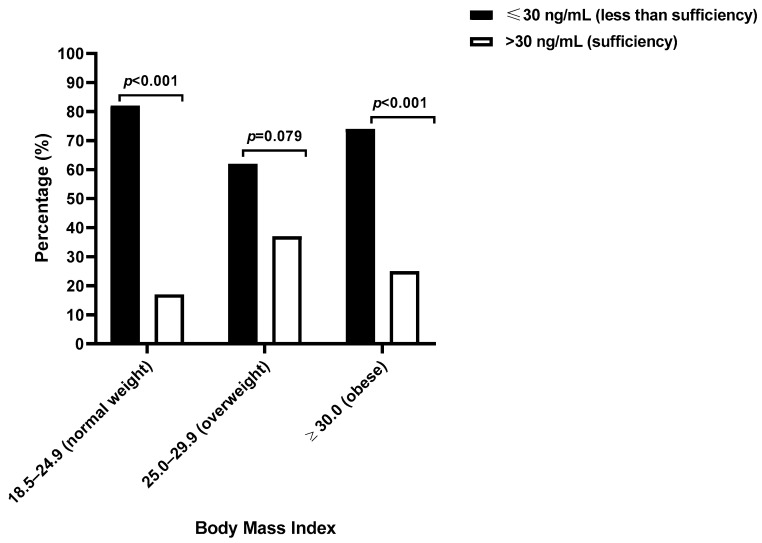
The vitamin D sufficient or less-than-sufficient levels according to body mass index among Eastern Cape women with cervical lesions.

Notably, women with normal BMI values were significantly more likely to have vitamin D levels of 30 ng/mL or lower than levels above 30 ng/mL (82.14%, 23/28 compared with 17.85%, 5/28; *p* < 0.001), a pattern also observed among obese women (74.42%, 32/43 compared with 25.58%, 11/43; *p* < 0.001) but not in overweight women (62.50%, 20/32 compared with 37.50%, 12/32; *p* = 0.079, Figure 2). Overweight women had approximately twice the likelihood of having sufficient vitamin D compared with women of a normal BMI (PR: 2.10, 95% CI: 0.89–5.22), although this difference was not statistically significant (*p* = 0.150).

## 4. Discussion

To our current knowledge, this study was among the first to describe the status of serum vitamin D and the associated factors among women with cervical lesions in the Eastern Cape population. A high burden of hypovitaminosis D, with 26.21% of women classified as deficient, 46.60% as insufficient, and only 27.18% reaching sufficiency, was observed. While this pattern aligns with the widespread vitamin D insufficiency reported across Sub-Saharan Africa [18], the finding that more than 70% of participants had more deficiency and insufficiency levels indicates that suboptimal vitamin D status is common in this population. Given the absence of a control group and the cross-sectional design, these findings should be interpreted as descriptive and hypothesis-generating, rather than indicative of causal relationships.

Vitamin D deficiency was observed across all age categories, and no statistically significant association between age and vitamin D status was identified. Although younger women showed a higher prevalence of deficiency, and a modest increase was also observed among the oldest age group, these differences did not reach statistical significance. This finding contrasts with observations from many high-income settings, where vitamin D levels tend to decline with an advancing age [19] and with reports of high deficiency rates among older adults in Western populations [20,21,22,23], but is consistent with reports where comparable observations have been noted, including stable vitamin D levels and lower odds of deficiency in older adults than younger adults [24,25,26], suggesting that age-related patterns of vitamin D status may vary across populations. In the present study, the widespread distribution of deficiency across age groups suggests that age alone may not be a dominant determinant of vitamin D status.

The overall low prevalence of vitamin D sufficiency (27.18%) among Eastern Cape women with cervical lesions and high HIV burden is disturbing, given its potential role in immune modulation, metabolic regulation, cellular proliferation, differentiation, and apoptosis and its potential influence on HIV and gynaecological disease outcomes [27,28,29]. In this study, we observed no statistically significant association between serum vitamin D status and HIV infection or cervical cytology/histology category among women. Vitamin D deficiency and insufficiency were prevalent across HIV-positive and HIV-negative participants as well as across different cytological classifications, indicating that a suboptimal vitamin D status was common irrespective of HIV status and cytology severity. Our finding of no significant difference in vitamin D status by HIV infection is consistent with other reports, where serum 25-hydroxyvitamin D concentrations did not differ markedly between people living with HIV on ART and HIV-negative individuals [30]. While HIV infection has been biologically linked to altered vitamin D metabolism partly due to immune activation and ART effects [31,32], the uniform ART exposure reported by all HIV-positive participants in our study may have attenuated differences between groups and limits direct comparison in this context.

No statistically significant association was identified between vitamin D status and cervical cytology/histology category. The results indicate that, within this study, serum vitamin D concentrations did not differ meaningfully according to cytological severity. These findings are consistent with reports from some studies indicating that vitamin D deficiency may be prevalent among women with cervical abnormalities without clear variation by lesion grade, particularly after accounting for immune and viral factors [33,34], However, recent studies suggest an inverse relationship between vitamin D status and cervical neoplasia risk [14], with the vitamin D endocrine system potentially protecting against early disease via the inhibition of abnormal proliferation, apoptosis induction, inflammation regulation, and HPV lesion clearance [35,36,37,38].

Socio-demographic factors were not statistically significant predictors of vitamin D deficiency; however, an increasing level of education was associated with a decreasing proportion of deficiency. The direction of these associations aligns with reports linking higher education and socioeconomic status to potentially healthier dietary behaviour and greater health literacy [20,39]. Lifestyle factors such as smoking and alcohol use showed no clear association with vitamin D status. While tobacco contains compounds that can disrupt vitamin D metabolism [40], their impact was not evident in this study. It is important to note that this could have been affected by the limited participants with smoking or alcohol consumption habits in this study. Reproductive factors were also unrelated to vitamin D levels despite the known vulnerability in women of childbearing age [41,42], This suggests that vitamin D deficiency was common across socioeconomic strata in this population and may reflect broader environmental or contextual factors rather than individual socio-demographic characteristics.

In humans, vitamin D is primarily synthesized in the skin via sunlight exposure and requires two hydroxylations, first in the liver to 25(OH)D, then in the kidney to the active 1,25(OH)_2_D [19,38,43]. Its status is influenced not only by total 25(OH)D levels but also by factors affecting bioavailability, including transport proteins such as vitamin-D-binding protein (DBP), with variations in DBP potentially altering the fraction of bioactive vitamin D and impacting physiological function and disease risk [44]. South African studies suggests that personal sun exposure, including duration and skin area exposed, predicts 25(OH)D levels and accounts for part of the observed variance [45]. However, in this investigation, outdoor activity was not associated with vitamin D levels. The lack of association with sun exposure may reflect limitations of the exposure assessment, which did not capture duration, time of day, clothing practices, or body surface area exposed. Furthermore, data collection occurred exclusively during the winter months (June–August), when ultraviolet radiation is reduced in South Africa, potentially limiting cutaneous vitamin D synthesis across the entire cohort and reducing variability between exposure groups. As a result, self-reported sun exposure may be unreliable due to factors such as clothing, shade-seeking, high melanin pigmentation, and occupational patterns [46]. Although not statistically significant, darker-skinned participants tended to have lower vitamin D levels, consistent with the inhibitory role of melanin on UVB-driven synthesis [47].

The dietary intake of vitamin-D-containing foods was not significantly associated with serum vitamin D status. Importantly, none of the participants reported taking vitamin D supplements. The observed serum 25-hydroxyvitamin D concentrations likely reflected habitual dietary intake and endogenous synthesis rather than supplemental sources. The relationship between dietary intake and serum 25(OH)D is consistent in the wider literature. Some studies have reported only weak or non-significant correlations between dietary vitamin D intake and serum levels, reflecting the reality that few foods naturally contain substantial vitamin D and that diet alone is often insufficient to maintain optimal status without adequate sunlight exposure or supplementation [48,49]. Furthermore, in populations such as rural South Africans, where vitamin D supplementation is uncommon and food fortification is limited [50,51], the relative contribution of diet may differ from settings with fortified foods or widespread supplement use. A study of South African women reported that foods are generally poor sources of vitamin D, and cutaneous synthesis remains the major contributor to serum levels in otherwise healthy adults [32].

Previous studies show that obesity is associated with vitamin D deficiency [52]. In this study, BMI showed a paradoxical pattern; women with normal BMI values had the highest prevalence of deficiency, while obese or overweight women had lower prevalence. This contrasts with the existing literature, where obesity is linked to lower vitamin D levels due to sequestration in adipose tissue and volumetric dilution [53,54]. Mechanistically, excess adiposity may disrupt vitamin D metabolism via the leptin-mediated down-regulation of activation pathways [55]. Conversely, vitamin D deficiency may contribute to adiposity by increasing parathyroid hormone activity and calcium-driven lipogenesis, indicating a potentially bidirectional relationship [54]. The paradoxical observation of higher vitamin D deficiency among women with normal BMI values may reflect reverse causation or confounding by disease severity. Weight loss, cachexia, co-existing infections, or increased metabolic demands in more clinically burdened women could plausibly influence both body composition and vitamin D status in this population. These findings highlight population-specific dynamics in the interplay between body composition and vitamin D status.

In parallel with biomarker-based research, emerging diagnostic approaches may enhance the evaluation of cervical squamous intraepithelial lesions. Cervical elastography, an ultrasound-based technique that assesses tissue stiffness, has shown potential in differentiating normal cervical tissue from precancerous and malignant lesions by capturing biomechanical changes associated with disease progression. Recent evidence suggests that elastography provides reproducible and quantitative measures that may complement cytology and histopathology, potentially reducing diagnostic subjectivity. Although further validation is required, particularly among women with squamous intraepithelial lesions, such approaches represent promising adjuncts for future studies aimed at improving early detection and risk stratification [56].

The outcomes of this study should be interpreted in the light of several methodological considerations. The cross-sectional design limits the ability to determine temporal or causal relationships between vitamin D status and associated factors. The current study did not include women with normal cervical cytology; this limited the comparison to this group and the ability to conclude whether the observed prevalence of vitamin D deficiency is specific to women with cervical lesionsor reflects broader population trends. It is recommended that further studies consider investigating women with all cervical cytology or histology grades. The modest sample size constrained statistical power and precluded multivariable analyses, increasing the possibility of residual confounding. Information on sun exposure and dietary intake was collected using self-reported measures, which may be subject to recall bias and misclassification. Sun exposure assessment was limited by the use of a crude proxy that did not account for duration, timing, clothing practices, or body surface area exposed.

Vitamin D status was assessed using total serum 25-hydroxyvitamin D concentrations without a measurement of vitamin-D-binding protein or free or bioavailable vitamin D, which may vary across populations and influence biological activity. Data collection occurred during the winter months, which may have contributed to lower vitamin D levels and limits generalizability to other seasons. Additionally, information on human papillomavirus infection, antiretroviral therapy regimens and durations, inflammatory markers, and dietary fortification exposure was unavailable, limiting the further exploration of biological pathways relevant to vitamin D metabolism and cervical disease. Despite these limitations, the paper provides valuable descriptive data on vitamin D status among women with cervical lesions and identifies important considerations for future research in similar settings.

## 5. Conclusions

This paper has described the serum vitamin D status and associated factors among women with cervical lesion attending a tertiary hospital in the Eastern Cape Province of South Africa. A high prevalence of vitamin D deficiency and insufficiency was observed, indicating that suboptimal vitamin D status is common in this clinical population. Vitamin D deficiency was evident across age groups and categories of abnormal cervix, reflecting a widespread burden rather than concentration within specific subgroups. Vitamin D deficiency decreased with an increasing BMI, highlighting a population-specific dynamic in the interplay between body composition and vitamin D status. Vitamin D deficiency or insufficiency was significantly more common than sufficiency among HIV-positive women only. The outcomes of this study should be interpreted in the light of several methodological considerations. Owing to the study’s cross-sectional design and lack of normal cytology participants, the findings are descriptive and exploratory, underscoring the need for longitudinal and comparative research to better define the role of vitamin D in cervical disease.

## Figures and Tables

**Table 1:** The demographic characteristics of the participants.

Variables	n/103	Percent (%)
**Age (years)**		
18–30	6	5.83
31–40	17	16.50
41–50	37	35.92
51–60	18	17.48
61–81	25	24.27
**Marital status**		
Single	55	53.40
Married	40	38.83
Widowed	7	6.80
No response	1	0.97
**Education level**		
Primary (Grade 1–7)	29	28.16
High school (Grade 8–12)	46	44.66
Tertiary	21	20.89
None	7	6.80
**Employed**		
Yes	16	15.53
No	87	84.47
**Monthly income**		
None	4	3.88
ZAR < 1000	9	8.74
ZAR 1000–3999	72	69.90
ZAR 4000–6999	9	8.74
ZAR 7000–9999	4	3.88
ZAR > 10,000	3	2.91
No response	2	1.94
**Smoking**		
Yes	8	7.77
No	94	91.26
No response	1	0.97
**Drink alcohol**		
Yes	7	6.80
No	88	85.44
Past	7	6.80
**Sexual debut**		
≤16 years	38	36.89
17–18 years	30	29.13
≥19 years	33	32.04
No response	2	1.94
**Lifetime sex partners**		
1	19	18.45
2	23	22.33
3–4	33	32.04
≥5	25	24.27
No response	3	2.91
**Have been pregnant**		
No	4	3.88
Yes 1 time	11	10.68
Yes 2 times	14	13.59
Yes ≥ 3 times	73	70.87
No response	1	0.97
**Vaginal itchiness or discharge history**		
Yes	44	42.72
No	58	56.31
No response	1	0.97
**Genital ulcer/wart history**		
Yes	7	42.72
No	95	56.31
No response	1	0.97
**HIV status**		
Positive	69	66.99
Negative	30	29.13
Unknown	4	3.88
**Cervical cytology/histology status**		
HSIL/ASC-H	61	59.22
Cervical cancer	32	31.07
ASCUS/LSIL	10	9.71
**Vitamin D levels**		
<20 ng/mL	27	26.21
20–30 ng/mL	48	46.60
>30 ng/mL	28	27.18
**Vitamin-D-containing foods**		
None	5	4.85
Daily	20	19.42
Weekly	43	41.75
Monthly	35	33.98
**Skin complexion**		
Dark	69	67.00
Light	34	33.00
**Days spent outdoors/week**		
Once	10	9.71
2–3 days	14	13.59
4–6 days	15	14.56
7 days	64	62.14
**BMI (kg/m^2^)**		
18.5–24.9 (Normal)	28	27.20
25.0–29.9 (Overweight)	32	31.10
≥30.0 (Obese)	43	41.70

**Table 2:** Distribution of serum vitamin D concentrations by HIV status among women with cervical lesions.

Vit D Levels	All Women (N = 103)	HIV-Negative, N = 30	HIV-Positive N = 69	PR (95% CI) *	*p*-Value *
	% (95% CI) n	% (95% CI) n	% (95% CI) n		
<20 ng/mL (deficiency)	26.21 (18.64–35.49) 27	26.67 (13.98–44.65) 8	26.09 (17.11–37.58) 18	0.98 (0.50–2.03)	>0.999
20–30 ng/mL (insufficiency)	46.60 (37.26–56.18) 48	36.67 (21.81–54.55) 11	52.17 (40.59–63.53) 36	1.42 (0.89–2.48)	0.191
≤30 ng/mL (deficiency/ insufficiency)	72.82 (63.48–80.51) 75	63.33 (45.45–78.19) 19	78.26 (67.07–86.47) 54	1.24 (0.95–1.75)	0.141
>30 ng/mL (sufficiency)	27.18 (19.49–36.52) 28	37.67 (21.81–54.55) 11	21.74 (13.53–32.93) 15	0.59 (0.32–1.15)	0.141
*p* value ^#^	**<0.0001**	0.070	**<0.0001**		

* Compares HIV-negative and HIV-positive women, with HIV-negative as the reference. Four participants had unknown HIV status. ^#^ compares deficiency/insufficiency Vitamin D and sufficiency groups. Bold *p*-values indicate significance.

**Table 3:** Factors associated with Vitamin D deficiency status among women with cervical lesions.

Variables	N	Vit-D Deficiency *n* (%)	PR (95% CI)	*p*-Value
**Age**				
18–30 years	6	3 (50.00)	reference	
31–40 years	17	6 (35.30)	0.71 (0.28–2.16)	0.643
41–50 years	37	7 (18.90)	0.38 (0.15–1.18)	0.127
51–60 years	18	3 (16.70)	0.33 (0.09–1.23)	0.139
61–81 years	25	8 (32.00)	0.64 (0.27–1.19)	0.638
**Marital status**				
Single	55	16 (29.10)	reference	
Married	40	7 (17.50)	0.60 (0.27–1.27)	0.231
Widowed	7	3 (42.90)	1.47 (0.51–3.15)	0.665
Separated/Divorced	1	1 (100.00)	3.44 (0.69–20.77)	0.304
**Education level**				
No education	7	3 (42.90)	reference	
Primary (Grade 1–7)	29	11 (37.90)	0.89 (0.39–2.60)	>0.999
High school (Grade 8–12)	46	10 (21.70)	0.51 (0.21–1.54)	0.343
Tertiary	21	3 (14.30)	0.33 (0.10–1.26)	0.144
**Employed**				
No	87	21 (24.10)	reference	
Yes	16	6 (37.50)	1.55 (0.71–2.10)	0.353
**Monthly income**				
No income	4	1 (25.00)	0.45 (0.08–1.75)	0.559
<ZAR 1000	9	5 (55.60)	reference	
≥ZAR 1000–3999	72	17 (23.60)	0.43 (0.23–0.97)	0.056
ZAR 4000–6999	9	3 (33.30)	0.60 (0.19–1.68)	0.637
ZAR 7000–9999	4	1 (25.00)	0.45 (0.08–1.75)	0.559
ZAR > 10,000	3	0 (0.00)	0.00 (0.00–1.24)	0.205
No response	2	0 (0.00)	0.00 (0.00–1.51)	0.455
**Smoking**				
No	94	25 (26.60)	reference	
Yes	8	2 (25.60)	0.94 (0.26–2.45)	>0.999
No response	1	0 (0.00)	0.00 (0.00–3.25)	>0.999
**Drink alcohol**				
No	88	24 (27.30)	reference	
Yes	7	2 (28.60)	1.05 (0.29–2.63)	>0.999
Past	7	1 (14.30)	0.52 (0.09–2.03)	0.671
**Sexual debut**				
≤16 years	38	9 (23.70)	reference	
17–18 years	30	10 (34.50)	1.41 (0.66–2.98)	0.424
≥19 years	33	8 (22.90)	1.02 (0.45–2.29)	>0.999
No response	2	0 (0.00)	0.00 (0.00–3.35)	>0.999
**Lifetime sex partners**				
1	20	4 (20.00)	reference	
2	23	6 (26.10)	1.30 (0.46–3.87)	0.728
3–4	34	6 (17.60)	0.88 (0.30–2.68)	>0.999
≥5	25	11 (26.20)	2.20 (0.90–5.91)	0.119
**Have been pregnant**				
No	4	1 (25.00)	reference	
Yes 1 time	11	1 (9.10)	0.36 (0.05–3.19)	0.476
Yes 2 times	14	6 (42.90)	1.71 (0.45- 10.01)	>0.999
Yes ≥3 times	73	19 (26.00)	1.04 (0.33–5.87)	>0.999
**Vaginal itchiness and/or discharge history**				
No	58	13 (22.40)	reference	
Yes	44	14 (31.80)	1.42 (0.75–2.68)	0.366
No response	1	0 (0.00)	0.00 (0.00–4.19)	>0.999
**Genital ulcer/wart history**				
No	95	24 (25.30)	reference	
Yes	7	3 (42.90)	1.70 (0.60–3.44)	0.378
No response	1	0 (0.00)	0.00 (0.00–3.44)	>0.999
**HIV status**				
Negative	30	8 (26.70)	reference	
Positive	69	18 (26.10)	0.98 (0.50–2.03)	>0.999
unknown	4	1 (25.00)	0.94 (0.16–3.45)	>0.999
**Cervical cytology/histology status**				
ASC-US/LSIL	10	2 (20.00)	reference	
HSIL/ASC-H	61	16 (26.50)	1.31 (0.45–4.87)	>0.999
CA Cervix	32	9 (28.10)	1.41 (0.45–5.40)	>0.999
**Skin complexion**				
Light	34	6 (17.60)	reference	
Dark	69	21 (30.40)	1.73 (0.81–3.91)	0.234
**Vitamin-D-containing food**				
None	5	0 (0.00)	0.00 (0.00–1.70)	0.289
Daily	20	6 (30.00)	reference	
Weekly	43	14 (32.60)	1.09 (0.52–2.47)	>0.999
monthly	35	7 (20.20)	0.67 (0.27–1.69)	0.513
**Days spent outdoors per week**				
Once	10	3 (30.00)	reference	
2–3 days	14	5 (35.70)	1.19 (0.40–3.90)	>0.999
4–6 days	15	5 (33.50)	1.11 (0.37–3.64)	>0999
7 days	64	14 (21.90)	0.73 (0.30–2.19)	0.687
**BMI (kg/m^2^)**				
18.5–24.9 (Normal)	28	13 (46.40)	reference	
25.0–29.9 (Overweight)	32	7 (21.90)	0.47 (0.22–0.98)	0.058
≥30.0 (Obese)	43	7 (16.30)	0.35 (0.16–0.75)	**0.008**

Bold *p*-values indicate significance.

## Data Availability

The raw data supporting the conclusions of this article will be made available by the authors on request.

## Abbreviations

The following abbreviations have been used in this manuscript:

HPV	Human papillomavirus
HIV	Human immunodeficiency virus
LSIL	Low-grade squamous intraepithelial lesion
ASC-H	Atypical squamous cells, cannot exclude a high-grade lesion
ASC-US	Atypical cells of undermined significance
HSIL	High-grade squamous epithelial lesion
BMI	Body mass index

## References

[1] Jeon S.M., Shin E.A. (2018). Exploring vitamin D metabolism and function in cancer. Exp. Mol. Med..

[2] Dallavalasa S., Tulimilli S.V., Bettada V.G., Karnik M., Uthaiah C.A., Anantharaju P.G., Nataraj S.M., Ramashetty R., Sukoche-va O.A., Tse E. (2024). Vitamin D in cancer prevention and treatment: A review of epidemiological, preclinical, and cellular studies. Cancers.

[3] Punchoo R., Dreyer G., Pillay T.S. (2023). 25-Hydroxycholecalciferol inhibits cell growth and induces apoptosis in SiHa cervical cells via autocrine vitamin D metabolism. Biomedicines.

[4] Zhou E., Bhoora S., Pillay T.S., Punchoo R. (2025). Induction of cell death and regulation of autocrine vitamin D metabolism in cervical cancer by physiological and GI20 doses of 25-hydroxycholecalciferol. Int. J. Mol. Sci..

[5] Kučan R., Soltirovska Šalamon A., Andronikov D., Benedik E. (2018). Dietary sources of vitamin D, vitamin D supplementation, and its bio-viability. Cent. Eur. J. Paediatr..

[6] Darling A.L. (2020). Vitamin D deficiency in western dwelling South Asian populations: An unrecognised epidemic. Proc. Nutr. Soc..

[7] World Health Organization (2020). WHO Antenatal Care Recommendations for a Positive Pregnancy Experience. Nutritional Interventions Update: Multiple Micronutrient Supplements During Pregnancy.

[8] Benedik E. (2021). Sources of vitamin D for humans. International Journal for Vitamin and Nutrition Research. Sources of vitamin D for humans. Int. J. Vitam. Nutr. Res..

[9] Wakeman M. (2021). A literature review of the potential impact of medication on vitamin D status. Risk Manag. Healthc. Policy.

[10] Gopalkrishnan K., Karim R. (2025). Addressing global disparities in cervical cancer burden: A narrative review of emerging strategies. Curr. HIV/AIDS Rep..

[11] Denny L., Kuhn L. (2017). Cervical cancer prevention and early detection from a South African perspective. S. Afr. Health Rev..

[12] Bolon J., Samson A., Irwin N., Murray L., Mbodi L., Stacey S., Aikman N., Moonsamy L., Zamparini J. (2023). An audit of adherence to cervical cancer screening guidelines in a tertiary-level HIV clinic. South. Afr. J. HIV Med..

[13] Porter V.L., Marra M.A. (2022). The drivers, mechanisms, and consequences of genome instability in HPV-driven cancers. Cancers.

[14] Punchoo R., Dreyer G., Pillay T.S. (2025). 25-hydroxycholecalciferol serum level shows an inverse relationship with high-grade uterine cervical dysplasia in HIV-uninfected black women in South Africa. J. Clin. Med..

[15] Bowden S.J., Doulgeraki T., Bouras E., Markozannes G., Athanasiou A., Grout-Smith H., Kechagias K.S., Ellis L.B., Zuber V., Chadeau-Hyam M. (2023). Risk factors for human papillomavirus infection, cervical intraepithelial neoplasia and cervical cancer: An umbrella review and follow-up Mendelian randomisation studies. BMC Med..

[16] Holick M.F., Binkley N.C., Bischoff-Ferrari H.A., Gordon C.M., Hanley D.A., Heaney R.P., Murad M.H., Weaver C.M. (2011). Evaluation, treatment, and prevention of vitamin D deficiency: An endocrine society clinical practice guideline. J. Clin. Endocrinol. Metab..

[17] Adeniyi O.V., Masilela C., George J.A. (2024). Prevalence of vitamin D deficiency and its association with cardiometabolic risk factors among healthcare workers in the Eastern Cape province, South Africa; cross-sectional study. Sci. Rep..

[18] Mogire R.M., Mutua A., Kimita W., Kamau A., Bejon P., Pettifor J.M., Adeyemo A., Williams T.N., Atkinson S.H. (2020). Prevalence of vitamin D deficiency in Africa: A systematic review and meta-analysis. Lancet Glob. Health.

[19] Holick M.F. (2017). The vitamin D deficiency pandemic: Approaches for diagnosis, treatment and prevention. Rev. Endocr. Metab. Disord..

[20] Ginter J.K., Krithika S., Gozdzik A., Hanwell H., Whiting S., Parra E.J. (2013). Vitamin D status of older adults of diverse ancestry living in the greater Toronto area. BMC Geriatr..

[21] Brouwer-Brolsma E.M., Vaes A.M., van der Zwaluw N.L., van Wijngaarden J.P., Swart K.M., Ham A.C., van Dijk S.C., Enne-man A.W., Sohl E., van Schoor N.M. (2016). Relative importance of summer sun exposure, vitamin D intake, and genes to vitamin D status in Dutch older adults: The B-PROOF study. J. Steroid Biochem. Mol. Biol..

[22] Carrillo-Vega M.F., García-Peña C., Gutiérrez-Robledo L.M., Pérez-Zepeda M.U. (2017). Vitamin D deficiency in older adults and its associated factors: A cross-sectional analysis of the Mexican health and aging study. Arch. Osteoporos..

[23] Giustina A., Bouillon R., Dawson-Hughes B., Ebeling P.R., Lazaretti-Castro M., Lips P., Marcocci C., Bilezikian J.P. (2023). Vitamin D in the older population: A consensus statement. Endocrine.

[24] Lips P., de Jongh R.T., van Schoor N.M. (2021). Trends in vitamin D status around the world. J. Bone Miner. Res. Plus.

[25] Hovsepian S., Amini M., Aminorroaya A., Amini P., Iraj B. (2011). Prevalence of vitamin D deficiency among adult population of Isfahan City, Iran. J. Health Popul. Nutr..

[26] Zhou Y., Qin S., Zhu Y., Xu P., Gu K. (2023). Inverse association between isoflavones and prediabetes risk: Evidence from NHANES 2007–2010 and 2017–2018. Front. Nutr..

[27] Mehta S., Giovannucci E., Mugusi F.M., Spiegelman D., Aboud S., Hertzmark E., Msamanga G.I., Hunter D., Fawzi W.W. (2010). Vitamin D status of HIV-infected women and its association with HIV disease progression, anemia, and mortality. PLoS ONE.

[28] Botros D., Somarriba G., Neri D., Miller T.L. (2012). Interventions to address chronic disease and HIV: Strategies to promote exercise and nutrition among HIV-infected individuals. Curr. HIV/AIDS Rep..

[29] Jiménez M.A., Martínez I., Medrano L.M., Fernández A., Resino S. (2018). Vitamin D in human immunodeficiency virus infection: Influence on immunity and disease. Front. Immunol..

[30] Mansueto P., Seidita A., Vitale G., Gangemi S., Iaria C., Cascio A. (2015). Vitamin D deficiency in HIV infection: Not only a bone disorder. BioMed Res. Int..

[31] Yin M., Stein E. (2011). The effect of antiretrovirals on vitamin D. Clin. Infect. Dis..

[32] Mwango S., Carboo J., Ellis C., Cockeran M., Mels C.M., Kruger H.S. (2021). The association between serum vitamin D and body composition in South African HIV-infected women. South. Afr. J. HIV Med..

[33] Troja C., Hoofnagle A.N., Szpiro A., Stern J.E., Lin J., Winer R.L. (2020). Serum concentrations of emerging vitamin D biomarkers and detection of prevalent high-risk HPV infection in mid-adult women. Cancer Epidemiol. Biomark. Prev..

[34] Troja C., Hoofnagle A.N., Szpiro A., Stern J.E., Lin J., Winer R.L. (2021). Understanding the role of emerging vitamin D biomarkers on short-term persistence of high-risk human papillomavirus infection among mid-adult women. J. Infect. Dis..

[35] Özgü E., Yılmaz N., Başer E., Güngör T., Erkaya S., İbrahim Yakut H. (2016). Could 25-OH vitamin D deficiency be a reason for HPV infection persistence in cervical premalignant lesions?. J. Exp. Ther. Oncol..

[36] Avila E., Noriega-Mejía B.J., González-Macías J., Cortes-Hernández U., García-Quiroz J., García-Becerra R., Díaz L. (2023). The preventive role of the vitamin D endocrine system in cervical cancer. Int. J. Mol. Sci..

[37] Khalili S.M., Rafiei E.H., Havaei M., Alizadeh L., Ghahremani F., Keshavarz Z., Montazeri A., Riazi H. (2024). Relationship between human papillomavirus and serum vitamin D levels: A systematic review. BMC Infect. Dis..

[38] Căpraru A.M., Ardelean S., Zurbău-Anghel N., Dărăban A.M., Munteanu M., Rusu A.I. (2025). Vitamin D as a preventive and therapeutic agent in cervical cancer: Insights from clinical studies. J. Exp. Pharmacol. Toxicol..

[39] Tønnesen R., Hovind P.H., Jensen L.T., Schwarz P. (2016). Determinants of vitamin D status in young adults: Influence of lifestyle, sociodemographic and anthropometric factors. BMC Public Health.

[40] Yang L., Zhao H., Liu K., Wang Y., Liu Q., Sun T., Chen S., Ren L. (2021). Smoking behavior and circulating vitamin D levels in adults: A meta-analysis. Food Sci. Nutr..

[41] Song S.J., Zhou L., Si S., Liu J., Zhou J., Feng K., Wu J., Zhang W. (2013). The high prevalence of vitamin D deficiency and its related maternal factors in pregnant women in Beijing. PLoS ONE.

[42] Hou W., Yan X.T., Bai C.M., Zhang X.W., Hui L.Y., Yu X.W. (2016). Decreased serum vitamin D levels in early spontaneous pregnancy loss. Eur. J. Clin. Nutr..

[43] Nimitphong H., Holick M.F. (2013). Vitamin D status and sun exposure in Southeast Asia. Dermato-Endocrinology.

[44] Lisowska-Myjak B., Skarżyńska E., Wróbel M., Mańka G., Kiecka M., Lipa M., Warzecha D., Spaczyński R., Piekarski P., Banaszewska B. (2023). Investigation of the changes in concentrations of vitamin D-binding protein and lactoferin in plasma and peritoneal fluid of patients with endometriosis. Int. J. Mol. Sci..

[45] George J.A., Norris S.A., van Deventer H.E., Pettifor J.M., Crowther N.J. (2014). Effect of adiposity, season, diet and calcium or vitamin D supplementation on the vitamin D status of healthy urban African and Asian-Indian adults. Br. J. Nutr..

[46] Norval M., Coussens A.K., Wilkinson R.J., Bornman L., Lucas R.M., Wright C.Y. (2016). Vitamin D status and its conse-quences for health in South Africa. Int. J. Environ. Res. Public Health.

[47] Hanel A., Carlberg C. (2020). Skin colour and vitamin D: An update. Exp. Dermatol..

[48] Black L.J., Burrows S.A., Jacoby P., Oddy W.H., Beilin L.J., Ping-Delfos W.C.S., Marshall C.E., Holt P.G., Hart P.H., Mori T.A. (2014). Vitamin D status and predictors of serum 25-hydroxyvitamin D concentrations in Western Australian adolescents. Br. J. Nutr..

[49] Cabral M., Araújo J., Lopes C., Barros H., Guimarães J.T., Severo M., Ramos E. (2018). Relationship between dietary vitamin D and serum 25-hydroxyvitamin D levels in Portuguese adolescents. Public Health Nutr..

[50] Cheteni P., Khamfula Y., Mah G. (2020). Exploring food security and household dietary diversity in the Eastern Cape Province, South Africa. Sustainability.

[51] Nxasana N., Oladimeji K.E., Pulido-Estrada G.-A., Apalata T.R. (2023). Prevalence of micronutrient deficiency among people living with HIV in selected rural districts of the Eastern Cape Province of South Africa. Nutrients.

[52] Vranić L., Mikolašević I., Milić S. (2019). Vitamin D deficiency: Consequence or cause of obesity?. Medicina.

[53] Vanlint S. (2013). Vitamin D and obesity. Nutrients.

[54] Pereira-Santos M., Costa P.d.F., Assis A.d., Santos C.d.S., Santos D.d. (2015). Obesity and vitamin D deficiency: A systematic review and meta-analysis. Obes. Rev..

[55] Tsuji K., Maeda T., Kawane T., Matsunuma A., Horiuchi N. (2010). Leptin stimulates fibroblast growth factor 23 expression in bone and suppresses renal 1α, 25-dihydroxyvitamin D3 synthesis in leptin-deficient ob/ob Mice. J. Bone Miner. Res..

[56] Mlodawski J., Plusajska J., Detka K., Swiercz G., Mlodawska M. (2025). Reproducibility of quantitative cervical strain elastography in nonpregnant patients and the effect of vaginal misoprostol on measured parameters. Sci. Rep..

